# MiR-279-3p regulates deltamethrin resistance through *CYP325BB1* in *Culex pipiens pallens*

**DOI:** 10.1186/s13071-021-05033-5

**Published:** 2021-10-12

**Authors:** Xixi Li, Shengli Hu, Hongbo Zhang, Haitao Yin, Huan Wang, Dan Zhou, Yan Sun, Lei Ma, Bo Shen, Changliang Zhu

**Affiliations:** 1 Department of Pathogen Biology, Nanjing Medical University, Nanjing, Jiangsu 211166 People’s Republic of China; 2 Department of Pathogen Biology, Nanjing University of Chinese Medicine, Nanjing, Jiangsu 210046 People’s Republic of China; 3 Department of Stomatology, Fifty People’s Hospital of Yuhang District, Hangzhou, Zhejiang 311199 People’s Republic of China

**Keywords:** MicroRNA, Cytochrome P450, Insecticide resistance, Mosquito

## Abstract

**Background:**

The overuse of insecticides to control insect vectors has promoted extensive insecticide resistance in mosquitoes. In this study, the functions of microRNA (miR)-279-3p and its target *CYP325BB1* in the regulation of deltamethrin resistance in *Culex pipiens pallens* was investigated.

**Methods:**

Quantitative real-time reverse transcription PCR was used to detect the expression levels of miR-279-3p and *CYP325BB1*. Then, the dual-luciferase reporter assay system, RNA interference, CDC bottle bioassay and Cell Counting Kit-8 (CCK-8) assay were used to explore the roles of these molecules in deltamethrin resistance both in vivo and in vitro.

**Results:**

The expression patterns of miR-279-3p and *CYP325BB1* were compared between deltamethrin-sensitive (DS-strain) and deltamethrin-resistant (DR-strain) mosquitoes. Luciferase activity was downregulated by miR-279-3p, the effect of which was ablated by a mutation of the putative binding site for *CYP325BB1*. In DR-strain mosquitoes, the expression of miR-279-3p was increased by microinjection and oral feeding of miR-279-3p agomir (mimic). *CYP325BB1* mRNA levels were downregulated, which resulted in a higher mortality of the mosquitoes in miR-279-3p mimic-treated groups. In the DS-strain mosquitoes, microinjection of a miR-279-3p inhibitor decreased miR-279-3p expression, whereas the expression of *CYP325BB1* was increased; the mortality of these mosquitoes decreased significantly. In addition, overexpression of pIB/V5-His-*CYP325BB1* changed the sensitivity of C6/36 cells to deltamethrin in vitro. Also in DR-strain mosquitoes, downregulation of *CYP325BB1* expression by microinjection of si-*CYP325BB1* increased mosquito mortality in vivo.

**Conclusions:**

These findings provide empirical evidence of the involvement of miRNAs in the regulation of insecticide resistance and indicate that miR-279-3p suppresses the expression of *CYP325BB1*, which in turn decreases deltamethrin resistance, resulting in increased mosquito mortality. Taken together, the results provide important information for use in the development of future mosquito control strategies.

**Graphical abstract:**

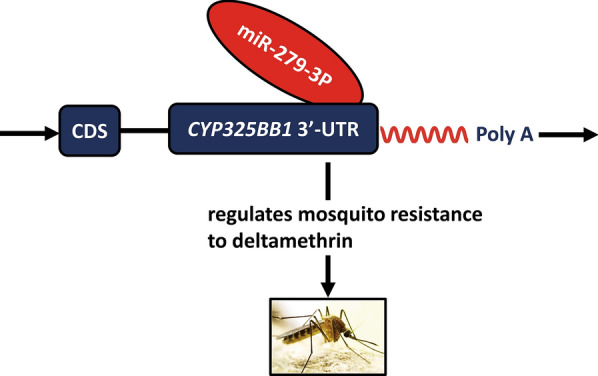

## Background

Mosquitoes are insect vectors of pathogens, including those causing serious human diseases, such as filariasis, encephalitis, West Nile fever, dengue fever and malaria, which threaten public health worldwide [1, 2]. Annually, insect-borne diseases cause illnesses in excess of half a billion people, of whom about 1 million die, including more than 400,000 children, one of whom dies from malaria every other minute of every day [3, 4]. Mosquito-borne diseases pose a growing threat to human health. Currently, the control of mosquito vectors is still an effective means to prevent and control mosquito-borne diseases. However, in recent decades, extensive insecticide use to control insect vectors has led to insecticide resistance, which is the main obstacle to mosquito control [5, 6]. Numerous studies have shown that increased metabolic detoxification of insecticides and decreased sensitivity of the target have resulted in complex multiple insecticide resistance [7]. Cytochrome P450 monooxygenases (P450s) have been shown to function in metabolic resistance by increasing the expression or activity of detoxification genes in mosquitoes [8]. Therefore, acquiring a detailed understanding of the molecular biology underlying insecticide resistance, particularly the posttranscriptional regulation of metabolic detoxification (especially for the P450s family), is important to develop novel approaches for pathogen and vector control [9].

MicroRNAs (miRNAs), which are noncoding RNAs of 22–23 nt, promote transcript decay and repress messenger RNA (mRNA) translation [10]. They bind to imperfect complementary sequences in the 3′ untranslated regions (3′-UTRs) of their target mRNAs [11]. In mosquito species, their particular developmental processes are affected by lineage-specific miRNAs, which might be developed as targets for vector control [12]. MicroRNAs interact with multiple target genes to elicit biological functions, and aberrant miRNA expression has been observed in development, metabolism, host–pathogen interactions and insecticide resistance. For example, in *Anopheles*, miR-276 affects the mosquito reproductive cycle and *Plasmodium falciparum* development via metabolic balancing [13]. The characterization and insecticide resistance functions of several mosquito miRNAs have recently been clarified [14–17]; however, the roles of mosquito-specific miRNAs and their participation in specific insecticide resistance events remain mostly unknown [18]. In insecticide-resistant mosquitoes, several miRNAs are dysregulated, suggesting that they may participate in insecticide resistance events. Our previous report on the high-throughput sequencing of the mosquito *Culex pipiens pallens* identified miR-279-3p as being highly expressed in deltamethrin-sensitive (DS-strain) mosquitoes compared deltamethrin-resistant (DR-strain) mosquitoes [19]; however, its role has remained undetermined.

The aim of the present study was to investigate the regulatory mechanism of deltamethrin resistance by determining the function of the miR-279-3p in the regulation of its target, *CYP325BB1* (cytochrome P450 325bb1). By analyzing the expression patterns of miR-279-3p and *CYP325BB1* in DR- and DS-strain mosquitoes subjected to RNA interference (RNAi), and function detection using CDC bottle bioassays, we detected a negative correlation between miR-279-3p and *CYP325BB1* with insecticide resistance in vivo. Cell viability analysis using the Cell Counting Kit-8 (CCK-8) assay validated the result in vitro. Thus, our data suggest that miR-279-3p might affect deltamethrin resistance by regulating *CYP325BB1* expression in *Cx. pipiens pallens*. These findings have important implications for understanding the mechanism of insecticide resistance and for developing mosquito control strategies in the future.

## Methods

### Mosquito strains and cell lines

Two strains of *Cx. pipiens pallens* with different resistance levels to deltamethrin were used in this study. The DS-strain of *Cx. pipiens pallens* used in this study was obtained from Ji Nan University and subsequently reared in our laboratory at 28 °C, 70–80% relative humidity, 14:10 h light/dark cycle. The DS-strain mosquitoes had not been exposed to any insecticides. The DR-strain was selected from the DS-strain by constant exposure to deltamethrin (at lethal concentration 50 [LC_50_]; concentration which kills 50% of sample population) and was screened over 80 generations. There were 4000 larvae screened for each pool (three pools/generation). The LC_50_ of the DS- and DR-strains was 0.05 and 8.5 mg/l, respectively. The resistance ratio of LC_50_ (RR_50_) of the DR-strain was 170. Deltamethrin (technical grade, 99.0%) was obtained from Jiangsu Provincial Center for Disease Control and Prevention (Jiangsu, China). Larvae were grown in dechlorinated tap water and fed with fish food powder (Tetramin; Tetra, Pirmasens, Germany) every 2 days. Adult mosquitoes were maintained in cages with constant access to a 5% glucose solution. Female mosquitoes were fed on mouse blood to reproduce the next generation every 3–4 weeks. Procedures for blood-feeding with mice in our laboratory were approved by The National Science and Technology of China and People’s Government of Jiangsu Province Animal Care and Use Committee and Institutional Review Board (No. IACUC-1812047).

The HEK293T cell line was grown in complete high glucose Dulbecco’s modified Eagle’s medium (DMEM; Gibco, Grand Island, NY, USA) containing 10% (v/v) fetal bovine serum (FBS; Gibco) and 100 U/ml penicillin–streptomycin solution (Gibco) at 37 °C under 5% CO_2_. Cells (6 × 10^4^/well) were seeded and incubated in 2.5 ml of complete growth medium in a 6-well plate for 24 h until they achieved > 80% confluency. The mosquito C6/36 cell line (CRL-1660; ATCC, Manassas, VA, USA) was cultured in DMEM supplemented with 10% FBS. The cells were plated in a 6-well plate and were grown in a 5% CO_2_-humidified incubator at 28 °C. Cells (5 × 10^5^ /well) were plated and incubated in 2.5 ml of complete growth medium in a 6-well plate for 24 h, until they achieved the required density of 60–80% for transfection. All cells were confirmed to be negative for mycoplasma contamination regularly.

### RNA and genomic DNA extraction

The RNAiso Plus reagent (Takara, Dalian, China) was used to extract total RNA from the female adult mosquitoes (*n* = 5) of the DS- and DR-strains at 3 days post-emergence (3 days PE). Genomic DNA (gDNA) was extracted from 3-day PE female adult mosquitoes using a MiniBEST Universal Genomic DNA Extraction Kit Ver. 5.0 (Takara) according to the manufacturer’s protocol. The quality and quantity of the RNA and gDNA were checked using a Thermo Scientific™ NanoDrop 2000 instrument (Thermo Fisher Scientific, Waltham, MA, USA).

### Quantitative real-time reverse transcription PCR analyses

A PrimeScript RT Reagent Kit (Takara) and PrimeScript™ RT Master Mix (Takara) were used to synthesize complementary DNA (cDNA) from 1 μg of total RNA following the manufacturer’s instructions. Then, 4 μl of 1:10 diluted cDNA solution was used as the template for quantitative real-time PCR (qPCR), which was performed using the Power SYBR Green PCR Master Mix (Applied Biosystems, Foster City, CA, USA). The PCR reaction mix (20 μl) contained forward and reverse PCR primers (10 pmol) for miR-279-3p, U6, *CYP325BB1* and *β-actin*, designed using Primer Premier 6.0 software (Premier Biosoft, Palo Alto, CA, USA; Table 1). Stem-loop reverse transcription (RT)-PCR was used to measure the expression of miR-279-3p [20]. The PCR cycling conditions were: 50 °C for 2 min; then 95 °C for 10 min; followed by 40 cycles of 95 °C for 15 s and 60 °C for 1 min; melting-curve analysis was then performed on an ABI Prism 7300 real-time PCR Instrument (Applied Biosystems). The miR-279-3p relative expression level was normalized to that of the internal control small nuclear RNA (U6), which is standard for miRNA expression normalization [21], and the expression level of *CYP325BB1* was normalized to that of *β-actin* from the DS- and DR-strains [22]. The DS-strain expression level was designated as 1, and the negative control (NC) was used to compare the gene expression levels. Each experiment used RNA from three biological replicates, and each cDNA sample was PCR amplified in triplicate. The 2^−ΔΔCt^ method was used to calculate the expression levels [23].

**Table 1:** Primers used for PCRs and vector constructions

Name of primer	Forward (5′–3′)	Reverse (5′–3′)
miR-279-3p (qRT-PCR)	ACACTCCAGCTGGGTGACTAGATCCACAC	TGGTGTCGTGGAGTCG
U6	GCTTCGGCTGGACATATACTAAAAT	GAACGCTTCACGATTTTGCG
*CYP325BB1*	TGCTGACCAGCGAACGAA	AGACCACCTTTCACCATCCC
*β-actin*	AGCGTGAACTGACGGCTCTTG	ACTCGTCGTACTCCTGCTTGG
*CYP325BB1* 3′-UTR-WT	TATCGGCTGTGGACTGACCTT	AAACCCATTTGGCATAAGACG
*CYP325BB1* 3′-UTR-Δ	TATCGGCTGTGGACTGACCTT	CCAAGCTTTTTTTGCCCACCAAGGTTT
pIB/V5-His-*CYP323BB1*	GGACTAGTGAGATGGAAATGCTGTTCGAAGTGCTCC	CCGCTCGAGCGTTTATTTCTCTTAGTCAACCAAACT

### Identifying the potential target of miR-279-3p

To identify the putative gene targets of miR-279-3p, we used 3′-UTR sequences from the *Culex quinquefasciatus* genome in the RNAhybrid target prediction program [24]. We focused on the cytochrome P450 family of genes (*CYP*) that participate in the regulation of insecticidal resistance of mosquitoes, and only *CYP325BB1* was identified as a potential target of miR-279-3p. To assess the conservation of the 3′-UTR, we amplified the 3′-UTR from *Cx. pipiens pallens*. The 3′-UTR sequence of *CYP325BB1* in *Cx. pipiens pallens* was 100% identical with that from *Cx. quinquefasciatus*.

### PMIR-REPORT vector construction and dual-luciferase reporter assay

To amplify the wild-type 3′-UTR (3′-UTR-WT) and the mutated 3′-UTR (3′-UTR-Δ) of *CYP325BB1* of *Cx. pipiens pallens*, primers for *CYP325BB1* 3′-UTR-WT/Δ (Table 1) were designed based on *Cx. quinquefasciatus* transcripts, which amplified the 3′-UTR region containing the miR-279-3p complementary sequences. Mutagenesis of the 3′-UTR (CTAGTCA) comprised replacing the WT binding site with CGACTGA. The *Cx. pipiens pallens CYP325BB1* 3′-UTR-WT and 3′-UTR-Δ sequences containing the putative seed region of the miR-279-3p binding sites were amplified and sequenced using the T/A cloning method. PCR products were run in 2.0% agarose gels and purified using a MiniBEST Agarose Gel DNA Extraction Kit Ver. 4.0 (Takara), and then cloned into vector pMD 19-T (Takara). The resultant plasmid was transferred into *Escherichia coli* One Shot® TOP10 Competent Cells (Invitrogen, Thermo Fisher Scientific, Carlsbad, CA, USA). The UTR sequences were then ligated into the HindIII and XbaI sites, which are located downstream of the *Renilla* translational stop codon in the pMIR-REPORT miRNA Expression Reporter Vector (Promega, Madison, WI, USA), to create the luciferase constructs. The pMIR-UTR-WT (6 ng) or pMIR-UTR-Δ were treated with miR-279-3p mimic (6 µl; GenePharma, Shanghai, China) and NC (GenePharma) along with PGL4.7 (6 ng; Promega), which were cotransfected using the FuGENE HD transfection reagent (Promega) into HEK293T cells (> 80% confluency). The Dual-Luciferase® Reporter Assay System (Promega) was then used for the reporter assay at 48 h after transfection. PGL4.7 provided the constitutive *Firefly* luciferase expression and was cotransfected as an internal control. *Renilla* luciferase was normalized to *Firefly* luciferase expression in each sample. The luciferase activity was detected at 560 and 480 nm using an M200 microplate fluorescence reader (Tecan, Lyon, France). The transfections were performed three times, and treatment was performed in triplicate.

### Microinjection

For the microinjection of miRNA, female adult mosquitoes were collected within 12 h PE and frozen at − 20 °C for 3–5 min. These mosquitoes were divided into three groups and prepared for injection through the thorax using a Nanoject III instrument (Drummond, Broomall, PA, USA). The miR-279-3p mimic or NC (0.5 μl; 20 nmol/l) were injected into the DR-strain mosquitoes (miR-279-3p mimic/NC group) under the same conditions. The miR-279-3p inhibitor or NC 1 (NC1, 0.5 μl; 20 nmol/l) was injected into the DS-strain mosquitoes (miR-279-3p inhibitor/NC1). For the microinjection of small interfering RNA (siRNA), 12-h PE DR-strain mosquitoes from the experimental group (si-*CYP325BB1*) and NC group were injected with 69 nl of si-*CYP325BB1* or NC (5 μg/μl) under the same conditions. Thereafter, the mosquitoes were maintained with a constant light/dark cycle (14/10 h) at 28 °C and 70–80% humidity. After 72 h, the expression levels of miR-279-3p and *CYP325BB1* were validated using qRT-PCR. Three biological replicates (each comprising 15 female mosquitoes) with three technical replicates were performed. GenePharma designed and produced all the injected RNA products (Table 2).

**Table 2:** Sequences of the miR-279-3p mimic, negative control, miR-279-3p inhibitor, inhibitor negative control 1 and si*-CYP325BB1*

Name	Sense (5′–3′)	Antisense (5′–3′)
miR-279-3p mimic	UGACUAGAUCCACACUCAUUA	AUGAGUGUGGAUCUAGUCAUU
Negative control (NC)	UUCUCCGAACGUGUCACGUTT	ACGUGACACGUUCGGAGAATT
miR-279-3p inhibitor	UAAUGAGUGUGGAUCUAGUCAC	
Inhibitor NC 1 (NC1)	CAGUACUUUUGUGUAGUACAA	
si*-CYP325BB1*	GGCUGGAAGUGCUCUUAAATT	UUUAAGAGCACUUCCAGCCTT

### Oral feeding

For the oral feeding experiments, pupae of the DR-strain were collected and placed in three cages until eclosion, and then fasted for 12 h. The mosquitoes of the control group were fed with 5% (w/v) glucose on a sponge wick (3 M, Minneapolis, MN, USA), while the NC group and the experimental group (miR-279-3p mimic) were given NC and miR-279-3p mimic (100 nmol/l) dissolved in 5% (w/v) glucose dissolved in DEPC-treated water, respectively. After 48 h of treatment, the RNAs of female adult mosquitoes were extracted to validate the expression levels of miR-279-3p and *CYP325BB1*. The miR-279-3p mimic and NC were obtained from GenePharma.

### U.S. Centers for Disease Control and Prevention bottle bioassay

To detect the mosquitoes’ sensitivity to deltamethrin after alteration of the miR-279-3p and *CYP325BB1* levels, U.S. Centers for Disease Control and Prevention (CDC) bottle bioassays were conducted according to published guidelines (https://www.cdc.gov/malaria/resources/pdf/fsp/ir_manual/ir_cdc_bioassay_en.pdf) [25]. Each bottle (250 ml) and its cap were rolled and inverted to coat them with 1 ml deltamethrin solution (7 mg/l for DR-strain; 0.01 mg/l for DS-strain). In parallel, a control bottle was coated with 1 ml of acetone, and then all bottles were left to dry in the dark. Mosquitoes from the designated groups (20/bottle) were exposed to deltamethrin or acetone for 2 h. Following exposure, the mosquitoes were monitored at 15-min intervals for 2 h and the percentage mortality (*y*-axis) was plotted against time (*x*-axis) using a linear scale.

### PIB/V5-His vector construction, transfection and detection

Standard molecular biology procedures were used for plasmid constructions. Standard overlap PCR was performed to amplify the open reading frame (ORF) of *CYP325BB1* using the pIB/V5-His-*CYP325BB1* primer pair (Table 1) from *Cx. quinquefasciatus*, which was ligated between unique restriction enzyme sites (SpeI/XhoI) of the eukaryotic expression vector pIB/V5-His. The positive recombinant plasmid was named pIB/V5-His-*CYP325BB1* and was confirmed using DNA sequencing.

C6/36 cells at 60–80% confluence were used for transfection. Plasmid DNA (pIB/V5-His-*CYP325BB1*) was diluted in complete growth medium to 1.5 ng/100 µl and 5 µl of FuGENE HD transfection reagent was added, followed by incubaion at room temperature with shaking for 25 min. pIB/V5-His was transfected as a control. Three biological replicates with three technical replicates were performed.

To evaluate the transfection efficiency of *CYP325BB1*, after 48 h of transfection, the mRNA and protein levels of CYP325BB1 in transiently transfected C6/36 cells were detected. Total RNA was isolated from the transfected cells and subjected to qRT-PCR to check the expression level of *CYP325BB1*. Protein was extracted from transfected cells, followed by washing in phosphate buffered saline (PBS), digestion in trypsin solution and lysis using radioimmunoprecipitation assay (RIPA) buffer (Beyotime, Jiangsu, China). The protein concentration was tested using a bicinchoninic acid (BCA) Protein Assay kit (Pierce, Rockford, IL, USA). Soluble protein (50 µg) was denatured and subjected to 10% sodium dodecyl sulfate-polyacrylamide gel electrophoresis. The proteins were transferred to a nitrocellulose membrane using a Trans-Blot System for 60 min at 300 mA (Bio-Rad, Hercules, CA, USA), and then washed twice in 1× Tris-buffered saline-Tween20 (TBS-T),and blocked for 60 min at 37 °C in 5% Difco™ Skim Milk (BD Biosciences, San Jose, CA, USA). The membrane was then incubated with anti-His-Tag monoclonal primary antibodies (1:1000; NovaGen, Madison, WI, USA) and anti-β-actin monoclonal primary antibodies (1:2000; ABGENT, Suzhou, China), with shaking overnight at 4 °C. Thereafter, the membranes were washed using TBS-T, and incubated with horseradish peroxidase (HRP)-conjugated goat anti-mouse secondary antibody (1:2000; Bioworld, Shenzhen, China) in blocking buffer at 37 °C for 2 h. The membranes were washed thoroughly using TBS-T, and the immunoreactive protein bands were visualized using Pierce™ ECL Western Blotting Substrate, before imaging using the Universal Hood Gel Doc System (Bio-Rad).

### C6/36 cell viability assay

The viability of cells overexpressing *CYP325BB1* was investigated using a CCK-8 kit assay (Dojindo, Kumamoto, Japan) [26]. C6/36 cells (100 µl) were added to each well of a 96-well plate at 5 × 10^3^ cells/well and incubated in a 5% CO_2_-humidified incubator at 28 °C for 24 h. At 24 h after transfection the cells were treated with 100 µl of deltamethrin at concentrations of 0, 10^0.5^, 10^1^, 10^1.5^, 10^2^ and 10^2.5^ mg/l [27]. After a further 24 h, the CCK-8 solution (10 µl) was added to each well and incubated at 28 °C for 3 h. The absorbance was then measured using a dual wavelength spectrophotometer in a microplate reader at 450 and 630 nm. Dimethyl sulfoxide (DMSO; Sigma, St. Louis, MO, USA) was used to dissolve deltamethrin at a final concentration of 0.5% (v/v). Three biological replicates with three technical replicates were performed.

### Statistical analysis

Statistically significant qualitative variables were detected using GraphPad Prism 8.0 software (GraphPad Software Inc., La Jolla, CA, USA). Data from independent experiments are presented as the mean ± the standard error of mean (SEM). Student’s t-test was used to determine the statistical significance of gene expression compared with that in the NC. The Chi-squared test (*χ*^2^) was used to analyze mosquito mortality. Statistical significance was indicated by *P* < 0.05 [28, 29]. All experiments were performed in at least three independent cohorts.

## Results

### MiR-279-3p targets *CYP325BB1*

**Fig. 1:**
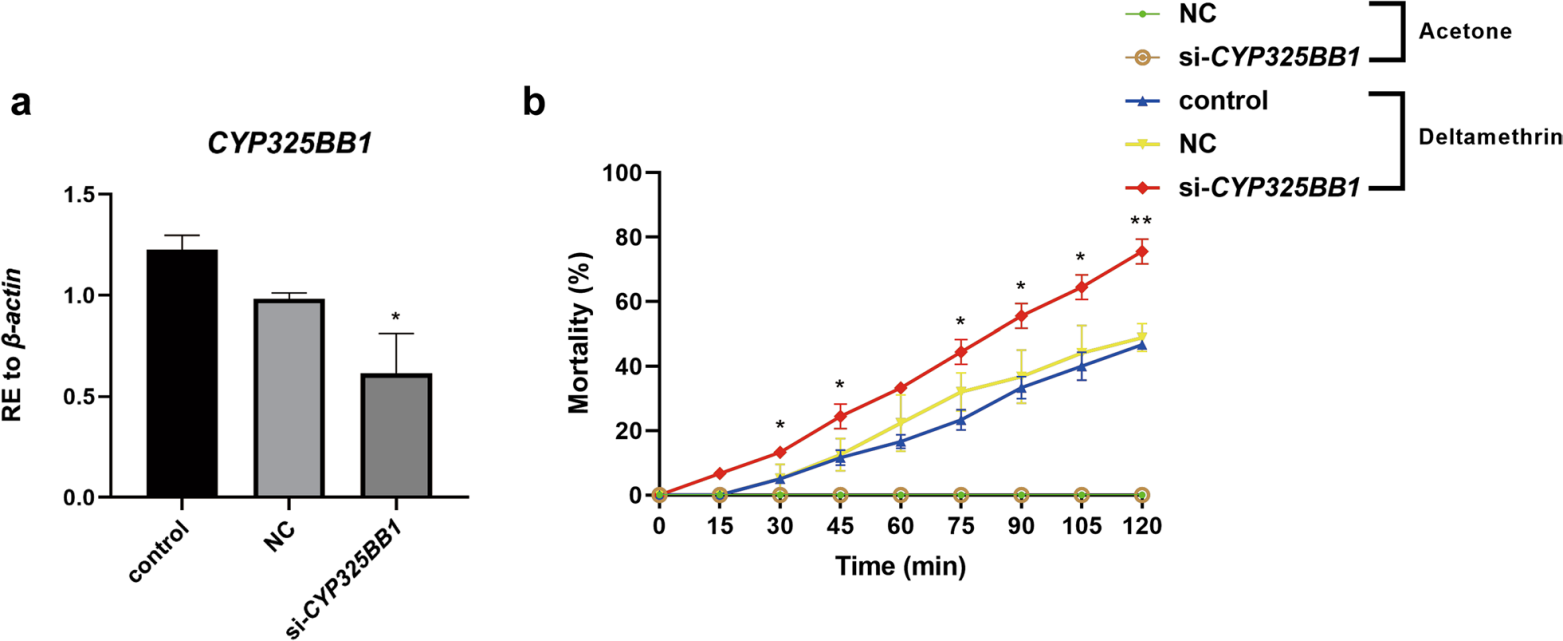
Transcription level of miR-279-3p and *CYP325BB1* in the DS-strain and DR-strain of *Culex pipiens pallens.* **a** MiR-279-3p expression was 2.97-fold higher in DS-strain than in DR-strain mosquitoes. **b** *CYP325BB1* expression was 2.31-fold greater in DR-strain than in DS-strain mosquitoes. Data are representative of three technical replicates of three biological replicates and are reported as the mean ± standard error of the mean (SEM). Asterisks indicate significant difference at *P < 0.05, ** P < 0.01. Abbreviations: RE, Relative expression, U6, internal control small nuclear RNA

Preliminary Solexa sequencing results showed that miR-279-3p expression was significantly different between DR- and DS-strain mosquitoes [19]. Then, qRT-PCR was used to assess miR-279-3p expression in the DR- and DS-strains. MiR-279-3p expression was 2.97-fold higher in the DS-strain than in the DR-strain (Fig. 1a; ***P < 0.001). In contrast, the expression of *CYP325BB1* was 2.31-fold higher in the DR-strain than in the DS-strain (Fig. 1b; ***P < 0.001). This contrasting expression pattern of miR-279-3p and *CYP325BB1* suggested that miR-279-3p might target *CYP325BB1*.

**Fig. 2:**
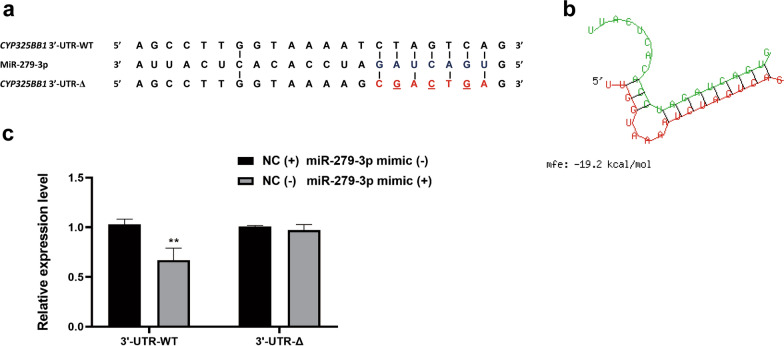
MiR-279-3p targets *CYP325BB1* via its 3′-UTR. **a** A region of the *CYP325BB1* 3′-UTR containing a putative binding site for miR-279-3p. **b** The free binding energy of *CYP325BB1* targeted by miR-279-3p predicted by the RNAhybrid software. **c** A dual-luciferase reporter assay demonstrated 33.0% decreased luciferase activity, suggesting that miR-279-3p could bind to the 3′-UTR of *CYP325BB1*. Data are representative of three technical replicates of three biological replicates and are reported as the mean ± SEM. Asterisks indicate significant difference at **P < 0.01

To verify this hypothesis, bioinformatic analysis was performed using the database of *Cx. quinquefasciatus* P450 sequences, which showed that the 3′-UTR of *CYP325BB1* harbored a miR-279-3p target site (Fig. 2a, b). To assess the regulatory relationship between the miRNA and the target mRNA, we performed a dual-luciferase reporter assay to detect the interaction between miR-279-3p and *CYP325BB1 *in vitro. The *CYP325BB1* 3′-UTR-WT and 3′-UTR-Δ were inserted into the *Renilla* translational stop codon in the pMIR-REPORT miRNA Expression Reporter vector, generating 3′-UTR-WT/Δ-fused luciferase reporters, which were cotransfected with control plasmid (PGL4.7) into HEK293T cells, and then treated with miRNA-279-3p mimic or NC. In the 3′-UTR-WT group, the luciferase intensity was inhibited by about 33.0%, showing that miR-279-3p could bind to the 3′-UTR construct, while there was no significant change in luciferase intensity in the NC group. Importantly, there was no change in luciferase intensity in the 3′-UTR-Δ group, whether treated with the miR-279-3p mimic or NC (Fig. 2c; **P < 0.01). Therefore, *CYP325BB1* was confirmed as a target gene of miR-279-3p in vitro.

### MiR-279-3p modulates deltamethrin resistance of mosquitoes

**Fig. 3:**
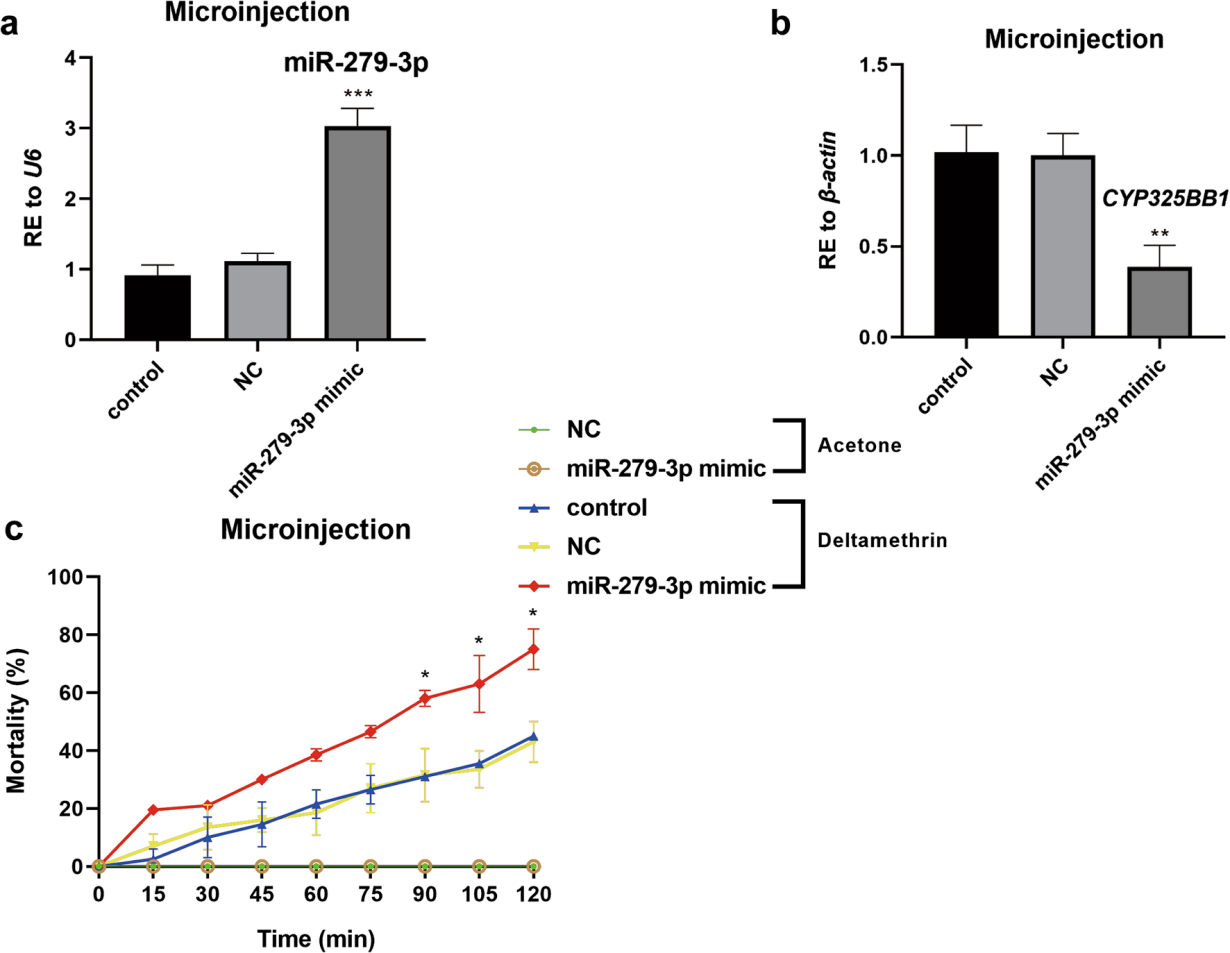
MiR-279-3p-mimic-injected DR-strain mosquitoes are more sensitive to deltamethrin. **a** Relative expression of miR-279-3p was 2.72-fold higher in the DR-strain mosquitoes at 72 h after injection with the miR-279-3p mimic, compared to the NC and control groups. **b** Relative expression of *CYP325BB1* was decreased by 61.3% in the DR-strain mosquitoes at 72 h after injection with the miR-279-3p mimic compared to the NC and control groups. **c** Mortality following 7 mg/l deltamethrin treatment of the miR-279-3p-mimic-injected group ranged from 57.4 to 74.5% at 90–120 min in the CDC bottle bioassay, which was higher than that in the acetone control, control and NC groups. Data are representative of three technical replicates of three biological replicates and are reported as the mean ± SEM. Asterisks indicate significant difference at *P < 0.05, **P < 0.01, ***P < 0.001. Abbreviations: RE Relative expression

To validate the participation of miR-279-3p in the regulation of deltamethrin resistance, mosquitoes overexpressing miR-279-3p were constructed. The miR-279-3p mimic was injected into 12-h PE DR-strain mosquitoes. The results showed that miR-279-3p was efficiently overexpressed (by 2.72-fold) in the miR-279-3p mimic injection group (Fig. 3a; ***P < 0.001). To further validate the regulatory relationship between miR-279-3p and *CYP325BB1 *in vivo, the transcription level of *CYP325BB1* was detected after overexpression of miR-279-3p in mosquitoes. Overexpression of miR-279-3p downregulated the expression of *CYP325BB1* by 61.3% (Fig. 3b; **P < 0.01). The results of the CDC bottle bioassay showed significantly higher mortality rates after the miR-279-3p mimic was injected compared with the control. After 105 min, the experimental group showed a mortality rate of 61.7% (29/47), whereas the NC group showed a mortality rate of 43.9% (18/41); in comparison, the control group showed a mortality rate of 40.0% (24/60). Furthermore, after 120 min, the mortality rates were 74.5% (35/47) in the experimental group, 48.8% (20/41) in the NC group, and 46.7% (28/60) in the control group (Fig. 3c; *P < 0.05).

**Fig. 4:**
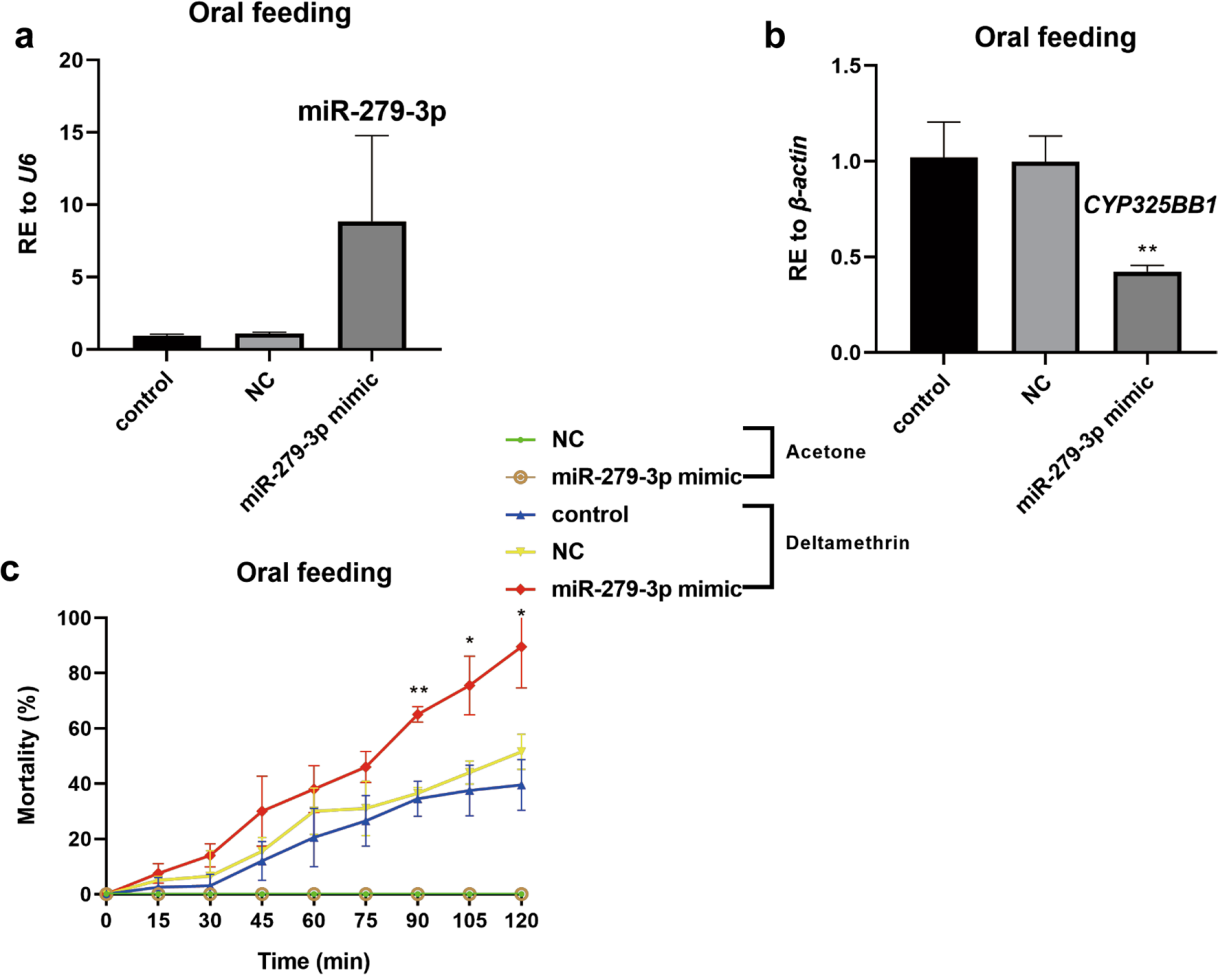
MiR-279-3p-mimic-supplied DR-strain mosquitoes by oral feeding reduced the intensity of deltamethrin resistance. **a** Relative expression of miR-279-3p was upregulated by 8.11-fold in the DR-strain mosquitoes at 48 h after oral feeding with the miR-279-3p mimic. **b** Relative expression of *CYP325BB1* was decreased by 57.5% in the DR-strain mosquitoes at 48 h after oral feeding with the miR-279-3p mimic. **c** Mortality of the miR-279-3p mimic group after incubation in 7 mg/l deltamethrin-coated CDC bottles for 2 h was higher than that in the acetone control, control and NC groups. The mortality rate ranged from 64.9 to 89.2% in the miR-279-3p mimic group at 90–120 min. Data are representative of three technical replicates of three biological replicates and are reported as the mean ± SEM. Statistical values are relative to the NC. Asterisks indicate significant difference at *P < 0.05, **P < 0.01. Abbreviations: RE, Relative expression

For the biocontrol of mosquitoes, in vivo delivery through feeding is required; therefore, the designed miR-279-3p mimic was supplied orally to mosquitoes. After ingestion, the relative expression of miR-279-3p was 8.11-fold higher than that in the NC group, which suggested that miR-279-3p was successfully overexpressed in the DR-strain (Fig. 4a). Consistently, in the miR-279-3p mimic oral feeding group, the expression of *CYP325BB1* was downregulated by 57.5% (Fig. 4b; **P < 0.01). In the CDC bottle bioassay, the miR-279-3p mimic feeding group experienced higher mortality than did the NC and control groups. After 90 min, the miR-279-3p mimic group showed a mortality rate of 64.9% (24/37), whereas the NC group showed a mortality rate of 42.4% (14/33) and the control group showed a mortality rate of 33.3% (11/33). Furthermore, after 120 min, the mortality rates were 89.2% (33/37), 57.6% (19/33) and 45.5% (15/33), respectively (Fig. 4c;*P < 0.05; **P < 0.01).

**Fig. 5:**
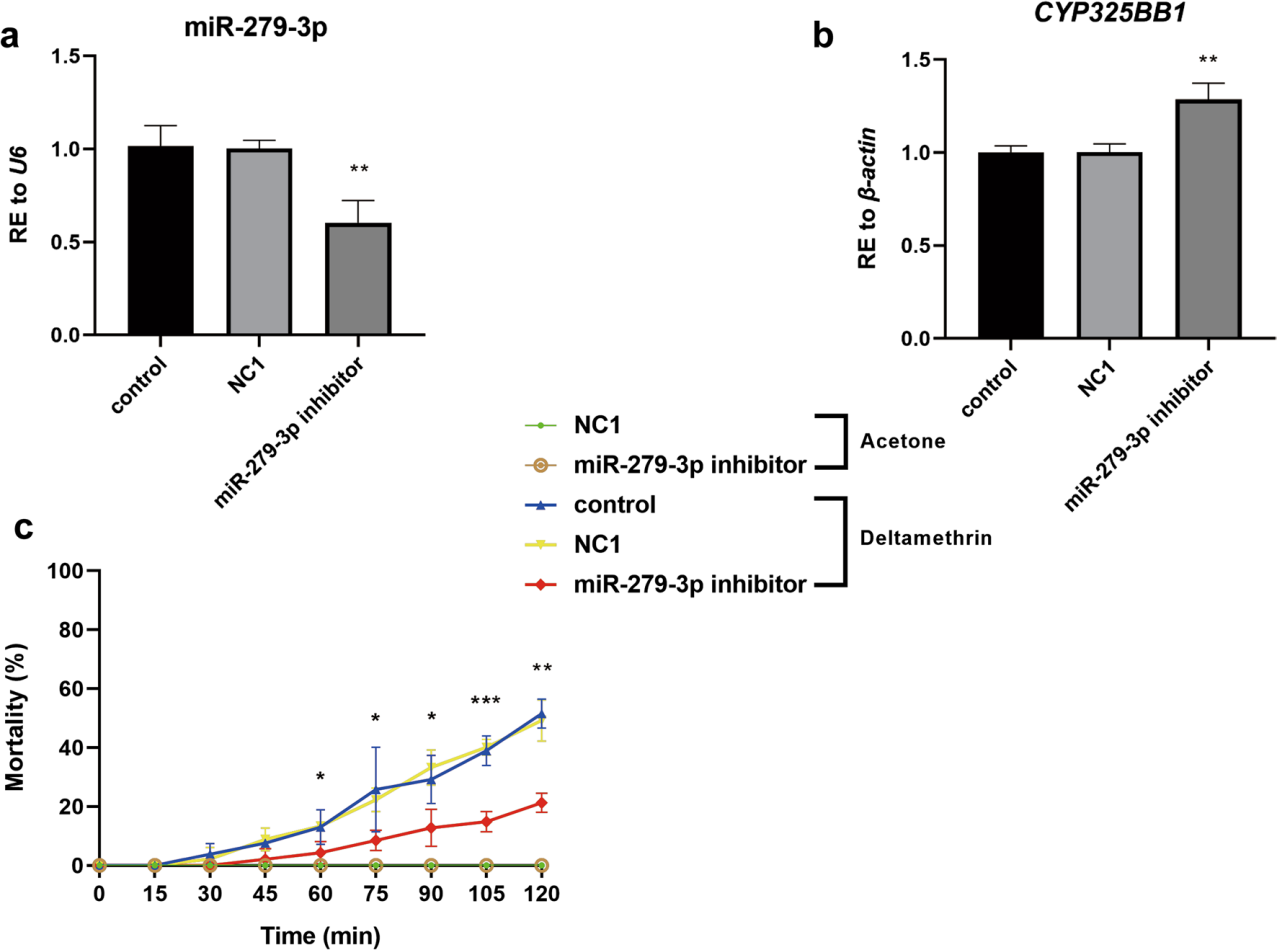
MiR-279-3p-inhibitor-injected DS-strain mosquitoes are more resistant to deltamethrin. **a** Relative expression of miR-279-3p was decreased by 40.2% in the DS-strain mosquitoes at 72 h after injection with the miR-279-3p inhibitor. **b** The relative expression of *CYP325BB1* was 1.28-fold higher in the DS-strain mosquitoes at 72 h after injection with the miR-279-3p inhibitor. **c** Mortality under 7 mg/l deltamethrin treatment of the miR-279-3p inhibitor-injected group ranged from 14.9 to 21.3% at 90–120 min in the CDC bottle bioassay, which was lower than that in the acetone control, control and NC1 groups. Data are representative of three technical replicates of biological replicates and are reported as the mean ± SEM. Asterisks indicate significant difference at *P < 0.05, **P < 0.01, ***P < 0.001. Abbreviations: NC1, Inhibitor negative control 1; RE, relative expression

Microinjecting the DS-strain mosquitoes with the miR-279-3p inhibitor was then performed. The relative expression of miR-279-3p decreased by 40.2% compared with that in the NC1 group (Fig. 5a; **P < 0.01) and, in contrast, the expression level of *CYP325BB1* increased by 1.28-fold compared with that in the NC1 group (Fig. 5b; **P < 0.01). The results of the CDC bottle bioassay showed significantly lower mortality rates after injection of the miR-279-3p inhibitor compared with that of the control. After 105 min, the experimental group showed a mortality rate of 14.9% (7/47), whereas the NC1 group showed a mortality rate of 40.0% (18/45), and the control group showed a mortality rate of 43.1% (22/51). Furthermore, after 120 min, the mortality rates were 21.3% (10/47) in the experimental group, 48.9% (22/45) in the NC1 group and 49.0% (25/51) in control group (Fig. 5c; *P < 0.05; ***P < 0.001). These results suggested that *CYP325BB1* is a bona fide target of miR-279-3p, that much higher expression of miR-279-3p is not intrinsically detrimental and that the miR-279-3p could modulate deltamethrin resistance by targeting *CYP325BB1* in mosquitoes.

### *CYP325BB1* functions in the deltamethrin resistance of mosquitoes

**Fig. 6:**
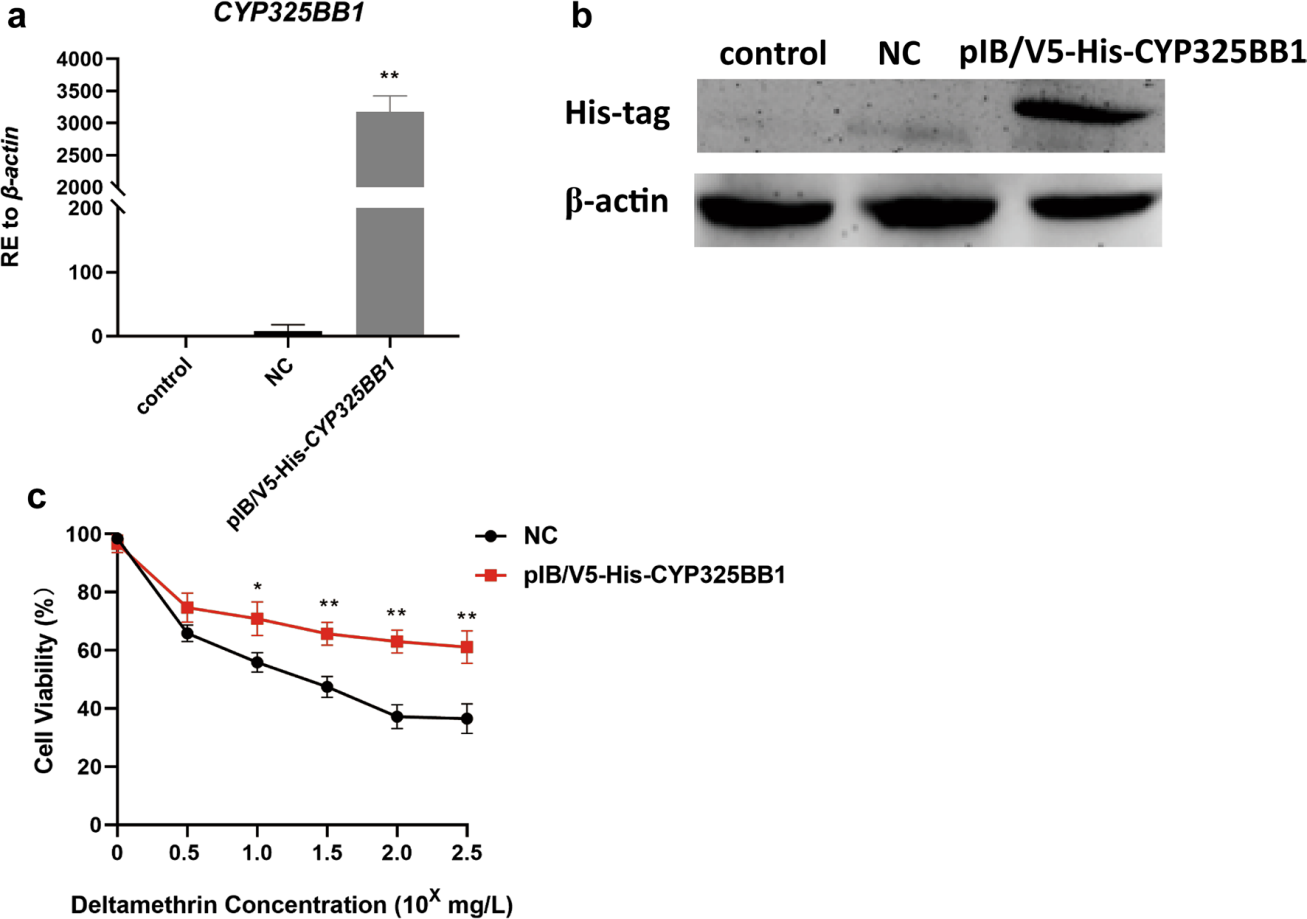
The viability of deltamethrin-treated C6/36 cells was upregulated after overexpression of *CYP325BB1* under stress. **a** Transcript level of *CYP325BB1* in the pIB/V5-His-*CYP325BB1-*transfected C6/36 cells increased by 397-fold compared with that in the NC group. **b** His-tag antibody detection of the levels of the CYP325BB1 protein. **c** Results of the Cell Counting Kit-8 (CCK-8) showed that the viability of pIB/V5-His-*CYP325BB1*-transfected C6/36 cells treated with deltamethrin was higher than that in the pIB/V5-His-transfected group. Data are representative of three technical replicates of biological replicates and are reported as the mean ± SEM. Asterisks indicate a significant difference at *P < 0.05, **P < 0.01. Abbreviation: NC, pIB/V5-His negative control; RE, relative expression

The regulatory role of *CYP325BB1* in mosquito resistance to deltamethrin in vitro was confirmed by amplifying the complete CYP325BB1 coding DNA sequence (GenBank: KM056313.1) from *Cx. pipiens pallens* and cloning it into the pIB/V5-His vector to generate plasmid pIB/V5-His-*CYP325BB1*. Total RNA was extracted from C6/36 cells of the control group, the pIB/V5-His NC group and the experimental group (pIB/V5-His-*CYP325BB1*). The results showed that the expression level of *CYP325BB1* was 397-fold higher than that in the NC group (Fig. 6a; **P < 0.01). Next, the protein expression level was validated using western blotting, and a band was detected using anti His-tag antibodies, which demonstrated that a protein of the expected size that was recognized by the anti-His-tag antibodies was expressed in the pIB/V5-His-*CYP325BB1-*transfected cells (Fig. 6b). Detection of the mRNA and protein levels proved that the transfection was successful. To investigate the sensitivity of the transiently transfected C6/36 cells to deltamethrin, a CCK-8 assay was employed to detect the viability of cells treated with deltamethrin in vitro. The proportion of viable cells was much higher among pIB/V5-His-*CYP325BB1*-transfected cells than cells in the NC group over a deltamethrin concentration range of 10^1^–10^2.5^ mg/l (Fig. 6c; *P < 0.05; **P < 0.01). Thus, the overexpression of *CYP325BB1* could increase cell resistance to deltamethrin.

**Fig. 7:**
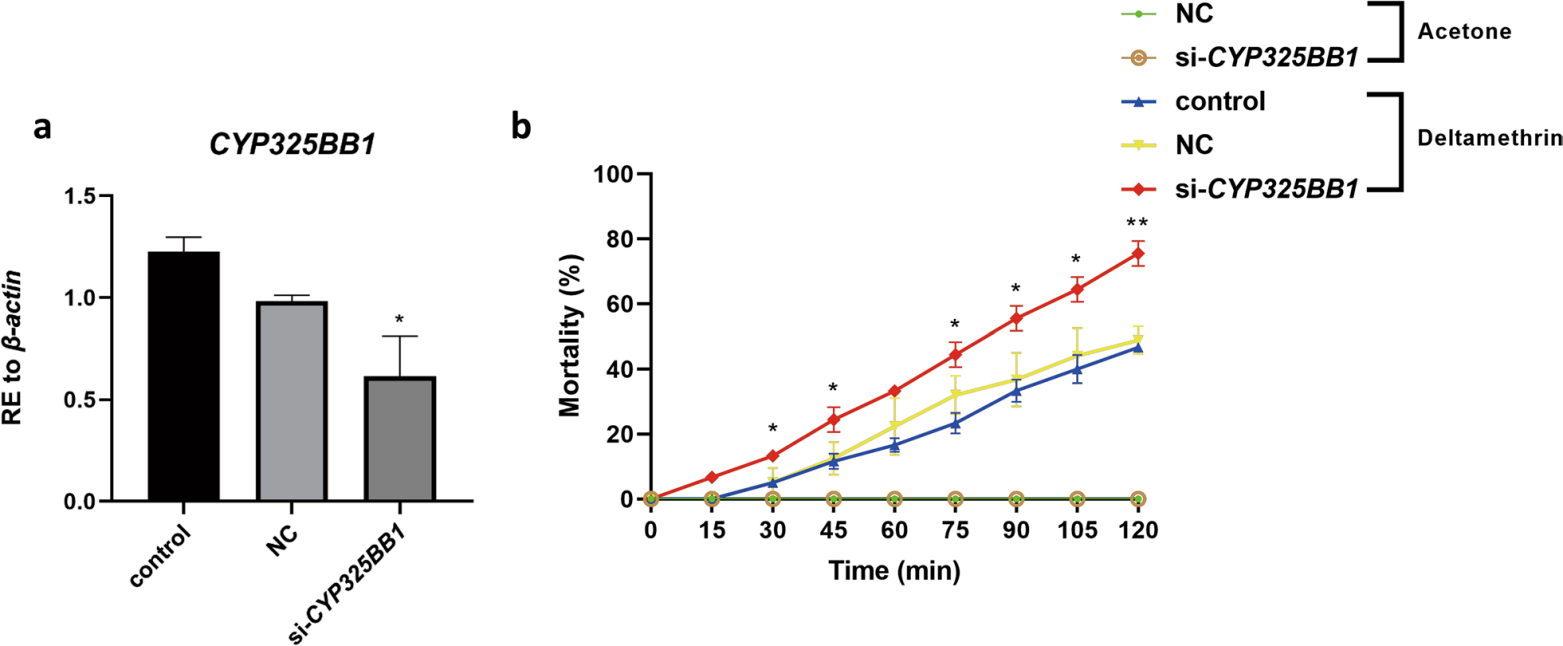
DR-strain mosquitoes injected with small interfering RNA targeting *CYP325BB1* (si-*CYP325BB1*) showed reduced deltamethrin resistance. **a** In the si-*CYP325BB1* injected DR-strain mosquitoes, *CYP325BB1* transcript levels were reduced by 37.6%. **b** Mortality of si-*CYP325BB1* group after incubation in CDC bottles coated with 7 mg/l deltamethrin for 2 h, was significantly higher than that in the acetone control, control and NC groups. Data are representative of three technical replicates of biological replicates and are reported as the mean ± SEM. Asterisks indicate significant difference at *P < 0.05, **P < 0.01. Abbreviations: RE, Relative expression; si, small interfering RNA

To further evaluate whether *CYP325BB1* participates in mosquito resistance to deltamethrin in vivo, we conducted phenotypic observation experiments after *CYP325BB1* RNAi knockdown (si-*CYP325BB1*) in DR-stain mosquitoes. *CYP325BB1* expression was 37.6% lower in the si-*CYP325BB1* group than in the control and NC groups (Fig. 7a; *P < 0.05). We then ran the CDC bottle bioassay to detect the mortality of si-*CYP325BB1*-injected mosquitoes. From 30 to 120 min, there was a significant difference in mortality, ranging from 13.3% (6/45) to 75.6% (34/45) in si-*CYP325BB1*-injected mosquitoes compared with the control (5.0% [3/60] to 46.7% [28/60]) and NC (4.9% [2/41] to 48.8% [20/41]) groups (Fig. 7b; *P* < 0.05, **P < 0.01). Thus, RNAi silencing of *CYP325BB1* in mosquitoes resulted in increased sensitivity to deltamethrin. Therefore, the RNAi of *CYP325BB1* produced the same phenotypes that were caused by overexpression of the miR-279-3p mimic.

Taken together, these results indicated that miR-279-3p might affect deltamethrin resistance by regulating *CYP325BB1* in *Cx. pipiens pallens*.

## Discussion

Mosquitoes and vector-borne diseases still represent a significant threat to human welfare; however, repeated exposures to insecticidal agents have caused high levels of resistance, representing an ongoing challenge to vector control [30]. Previous studies have provided evidence supporting the view that different mechanisms and genes are associated with the development of insecticide resistance [31]. MicroRNAs are short, well-characterized, non-coding, post-transcriptional gene regulators [32]. During the process of transforming primary precursors (pri-miRNAs) to pre-miRNAs, multiple regulatory proteins are involved that bind directly to distinct pre-miRNA in a sequence- or structure-dependent manner, to produce mature miRNAs [33]. Accordingly, miRNAs have been associated with a wide range of fundamental biological processes and are implicated in development, metabolism and host–pathogen interactions; indeed, many miRNAs are downregulated in the process of insecticide resistance [34]. In *Drosophila melanogaster* and *Plutella xylostella *(L.), changes in miRNA expression are induced by insecticides [35, 36]. Comparative and correlative studies have revealed that miRNAs are involved in the regulation of deltamethrin insecticide resistance; however, we lack strong evidence of miRNAs as causative factors of resistant phenotypes [14, 15, 37, 38].

Our previous high-throughput sequencing study identified miR-279-3p as being expressed at a significantly lower level in DR-strain mosquitoes compared to DS-strain mosquitoes [19]. The results of the present study demonstrated lower expression of miR-279-3p in the DR-strain compared with that in the DS-strain, which is consistent with the sequencing results. Certain miRNAs appear to show plasticity in transcription in the presence of pesticides in *Cx. pipiens pallens*, and certain miRNAs or miRNA clusters are downregulated in the DR-strain, such as miR-2 ~ 13 ~ 17, miR-4448 and miR-278-3p [14, 16, 17]. However, other miRNAs are upregulated in the DR-strain, such as miRNA-92a, miRNA-932 and miR-285 [15, 38, 39].

The miRNA we investigated is constitutively downregulated in the DR-strain. The miR-279-3p-mimic-injected DR-strain mosquitoes became more susceptible to deltamethrin, while the miR-279-3p-inhibitor-injected DS-strain mosquitoes became more resistant to deltamethrin, which validated that miR-279-3p participates in the regulation of deltamethrin resistance. The convenient, effective and environmentally friendly use of the miR-279-3p mimic as a molecular biopesticide in the future requires would require its oral feeding [40]. Therefore, the miR-279-3p mimic was delivered orally to induce RNA interference in the DR-strain mosquitoes. The mortality was higher in the miR-279-3p-mimic-fed group than in the NC group, which further confirmed miR-279-3p-induced regulation of mosquito resistance. According to previous reports, *E. coli* and *Saccharomyces cerevisiae* expression systems could be used for silencing target genes. The bioinsecticide could be used as an insecticidal adjuvant agent, thereby minimizing the environmental impact. However, further studies are necessary to establish the conditions for expression and large-scale culture, and to determine the effectiveness of this bioinsecticide via different forms of administration, such as spraying, in all stages of vector development. In addition, the use of this expression system in field applications should be regulated by the appropriate authorities in each country [41, 42].

MiRNA genes are encoded within the genome. Their transcription might be tightly coordinated with the transcription of other genes that serve either as a source of miRNAs or as their targets, including the protein-coding genes to exert miRNA biogenesis functions [43]. In support of this model, intronic miR-208a, which is coexpressed with its heart-specific host gene, *Myh6*, controls the suppression of negative regulators of muscle growth and hypertrophy in mice [44]. Recent studies on miRNA-mediated metabolic insecticide resistance have revealed that aberrant expression of miRNAs can induce marked changes in the expression levels of detoxification genes, especially those encoding cytochrome P450s [14, 37, 45]. Therefore, we firstly identified *CYP325BB1* as the miR-279-3p’s direct target in vitro and in vivo. In the dual-luciferase reporter assay, cotransfection of the luciferase reporter vector comprising the *CYP325BB1* 3′-UTR together with the miR-279-3p mimic caused a decrease in *Renilla* luciferase activity in vitro, and the overexpression of miR-279-3p reduced the *CYP325BB1* expression level in vivo. Taken together, these results confirmed *CYP325BB1* as an authentic target of miR-279-3p in mosquitoes.

Studies have demonstrated that increased expression of cytochrome P450s could enhance metabolic detoxification of insecticides in resistant mosquitoes [46, 47]. Thus, we investigated the mechanism of *CYP325BB1’*’s contribution to insecticide resistance following the functional test of *CYP325BB1 *in vitro and in vivo. The CCK-8 assay was used to detect C6/36 cell viability in vitro because *CYP325BB1* expression showed that cell viability was higher, indicating that this cytochrome is involved in deltamethrin resistance. Additionally, we injected si-*CYP325BB1* into mosquitoes and detected deltamethrin resistance in vivo. Our results showed higher mortality in the *CYP325BB1*-silenced group, indicating that reduced *CYP325BB1* expression abrogated deltamethrin resistance.

The findings of the present study demonstrate a link between decreased miR-279-3p expression and a deltamethrin resistance phenotype via post-transcriptional suppression of *CYP325BB1*, whose protein product is involved in xenobiotic detoxification. Therefore, miR-279-3p could represent a novel biomarker of insecticide exposure, and is one of the factors that could explain the insecticide’s toxic effects. These insights are important to understand how mosquitoes have adapted at the molecular level to evolve deltamethrin resistance, and highlight the importance of gaining a complete understanding of insect biology for insecticide studies.

## Data Availability

All data generated or analyzed during this study are included in this published article.

## Abbreviations

CDC: U.S. Centers for Disease Control and Prevention
CYP: Cytochrome P450
DR-strain: Deltamethrin-resistant strain
DS-strain: Deltamethrin-sensitive strain
miRs: MicroRNAs
NC: Negative control
qRT-PCR: Quantitative real-time reverse transcription PCR
UTR: Untranslated region
WT: Wild-type
